# Physics-based RNA structure prediction

**DOI:** 10.1007/s41048-015-0001-4

**Published:** 2015-07-09

**Authors:** Xiaojun Xu, Shi-Jie Chen

**Affiliations:** Department of Physics, University of Missouri, Columbia, MO 65211 USA; Department of Biochemistry, University of Missouri, Columbia, MO 65211 USA; Informatics Institute, University of Missouri, Columbia, MO 65211 USA

**Keywords:** RNA, Vfold, 2D structure prediction, 3D structure prediction, Tertiary motif

## Abstract

Despite the success of RNA secondary structure prediction for simple, short RNAs, the problem of predicting RNAs with long-range tertiary folds remains. Furthermore, RNA 3D structure prediction is hampered by the lack of the knowledge about the tertiary contacts and their thermodynamic parameters. Low-resolution structural modeling enables us to estimate the conformational entropies for a number of tertiary folds through rigorous statistical mechanical calculations. The models lead to 3D tertiary folds at coarse-grained level. The coarse-grained structures serve as the initial structures for all-atom molecular dynamics refinement to build the final all-atom 3D structures. In this paper, we present an overview of RNA computational models for secondary and tertiary structures’ predictions and then focus on a recently developed RNA statistical mechanical model—the Vfold model. The main emphasis is placed on the physics behind the models, including the treatment of the non-canonical interactions in secondary and tertiary structure modelings, and the correlations to RNA functions.

## Introduction

The increasing discoveries of noncoding RNAs demand more than ever the information about RNA structure (Bachellerie et al. 2002; Kertesz et al. 2007; He et al. 2008; Bartel 2009; Gong and Maquat 2011; Wang et al. 2013). For example, the 3D structure of a microRNA–target complex is crucial for understanding microRNA’s binding affinity and efficacy in gene regulation (Kertesz et al. 2007; Bartel 2009). However, the time-consuming, laborious, expensive experimental determination, such as X-ray crystallographic and NMR spectroscopic measurements, alone cannot catch up the pace with the rapidly increasing number of biologically significant RNAs such as noncoding regulatory RNAs. This problem highlights the need for computational prediction of RNA folding.

The structure of an RNA is determined by the complex pattern of base–base interactions, including base-paired secondary structures and long-range tertiary interactions. Existing RNA folding theories mainly focus on the secondary structures. However, knowing the secondary structure information alone is not sufficient to determine the 3D structure, because a 3D structure often involves long-range tertiary interactions such as kissing interactions between the different loops. Therefore, for a physics-based approach, accurate evaluation of the energetic parameters for tertiary interactions is critical for 3D structure prediction. Moreover, RNA function is correlated not only to the minimum free energy state of an RNA, but also to the potentially large conformational changes it can undergo. Understanding RNA function requires the understanding of the full energy landscape.

Statistical mechanics-based modeling has led to significant success in RNA structure prediction, folding stabilities, and folding kinetics for structures with different structural complexities (Liu et al. 2006; Chen 2008). For example, a recently developed statistical mechanics-based RNA folding model, “Vfold” model, has provided a wide range of quantitative predictions and novel insights for a variety of experiments and RNA functions, such as the pseudoknot-involved conformational switch between bistable secondary structures (Xu and Chen 2012), microRNA gene regulation through microRNA/mRNA-binding interactions (Cao and Chen 2012), and RNA/RNA dimerization critical for viral replication (Cao and Chen 2011; Cao et al. 2014). However, despite the success of this approach, several key issues remain. Estimation of the entropies for RNA tertiary folds and extraction of the energy parameters for noncanonical tertiary interactions from thermodynamic data or known structures present major challenges hampering the structural modeling for large and complex RNAs. The primary focus of this article is on the statistical mechanics-based methods for predicting RNA 3D structures and folding energy landscapes, and the related quantitative insights into RNA functions.

## An overview of computational methods for RNA folding

RNA folding process is believed to be partly hierarchical, whereby secondary structural motifs fold first followed by the tertiary contacts formation. The secondary structure is a set of helices containing canonical base pairs (A–U, G–C, and G–U) and contributes to the major part of the free energy of an RNA system. Canonical base pairing and base stacking within helices are generally stronger than the non-canonical interactions in loop parts of an RNA system. Therefore, many computational models dissect the RNA folding problem into two steps: from sequence to two-dimensional (2D) structure and from 2D structure to three-dimensional (3D) structure, where a 2D structure is defined by base pairs including tertiary cross-linked base pairs such as kissing base pairs. With the 2D structure as constraint, the accuracy of 3D structure prediction can be significantly improved.

### 2D structure predictions

Computational models for RNA 2D structure prediction fall into two general categories: free energy minimization (Ding and Lawrence 2003; Hofacker 2003; Zuker 2003; Xayaphoummine et al. 2005; Mathews and Turner 2006; Parisien and Major 2008; Bellaousov et al. 2013; Xu et al. 2014), and sequence comparison (Gutell et al. 2002; Hofacker et al. 2002; Mathews and Turner 2002; Havgaard et al. 2005; Bindewald and Shapiro 2006; Bernhart et al. 2008; Sato et al. 2009). Most free energy minimization methods employ the empirical thermodynamic parameters [the Turner parameters (Turner and Mathews 2010)] for the different secondary structural elements. Other models, such as MC-Fold (Parisien and Major 2008), use knowledge-based energy functions extracted from the known PDB structures. However, not all the interactions (such as long-range tertiary contacts) can be captured by these parameters. As a result, the accuracy of prediction falls off rapidly with the length of the sequence, because larger RNAs are more prone to the formation of long-range tertiary contacts.

The accuracy of computational predictions is usually better for methods that consider “fold recognition”: structure is usually more conserved than sequence, and the functional core regions are usually more conserved at all levels. In general, sequence comparison-based methods can give more improved predictions than free-energy-based predictions if the homologous sequences are available. However, these methods depend strongly on the availability of the sequence database. To overcome the above limitations, several hybrid algorithms that combine free energy minimization and sequence comparison have been developed (Mathews and Turner 2002; Havgaard et al. 2005; Bernhart et al. 2008). For example, Dynalign (Mathews and Turner 2002) combines free energy minimization and comparative sequence analysis to find a low free energy structure common to two sequences without requiring any sequence identity. On average, Dynalign predicted 86.1 % of known base-pairs in the tRNAs, compared to 59.7 % by free energy minimization alone. For the 5S rRNAs, the average accuracy improves from 47.8 to 86.4 %.

Another way to improve the accuracy of structure prediction is to incorporate experimental data to the secondary structure prediction modeling (Mathews et al. 2004; Deigan et al. 2009; Low and Weeks 2010; Kladwang et al. 2011; Hajdin et al. 2013; Leonard et al. 2013). Selective 2′-hydroxyl acylation analyzed by primer extension (SHAPE) probing data has proved useful for RNA secondary structure modeling (Deigan et al. 2009; Low and Weeks 2010; Kladwang et al. 2011; Hajdin et al. 2013; Leonard et al. 2013). The SHAPE information provides refinements for the experimental determined thermodynamic parameters (Turner and Mathews 2010) for RNA folding. Benchmark test for a set of 21 RNAs of size from 34 to 530 nt shows that 93 % on average of known base pairs can be predicted. and all pseudoknots in well-folded RNAs can be identified (Hajdin et al. 2013).

### Tertiary motifs: free energy models

**Fig. 1:**
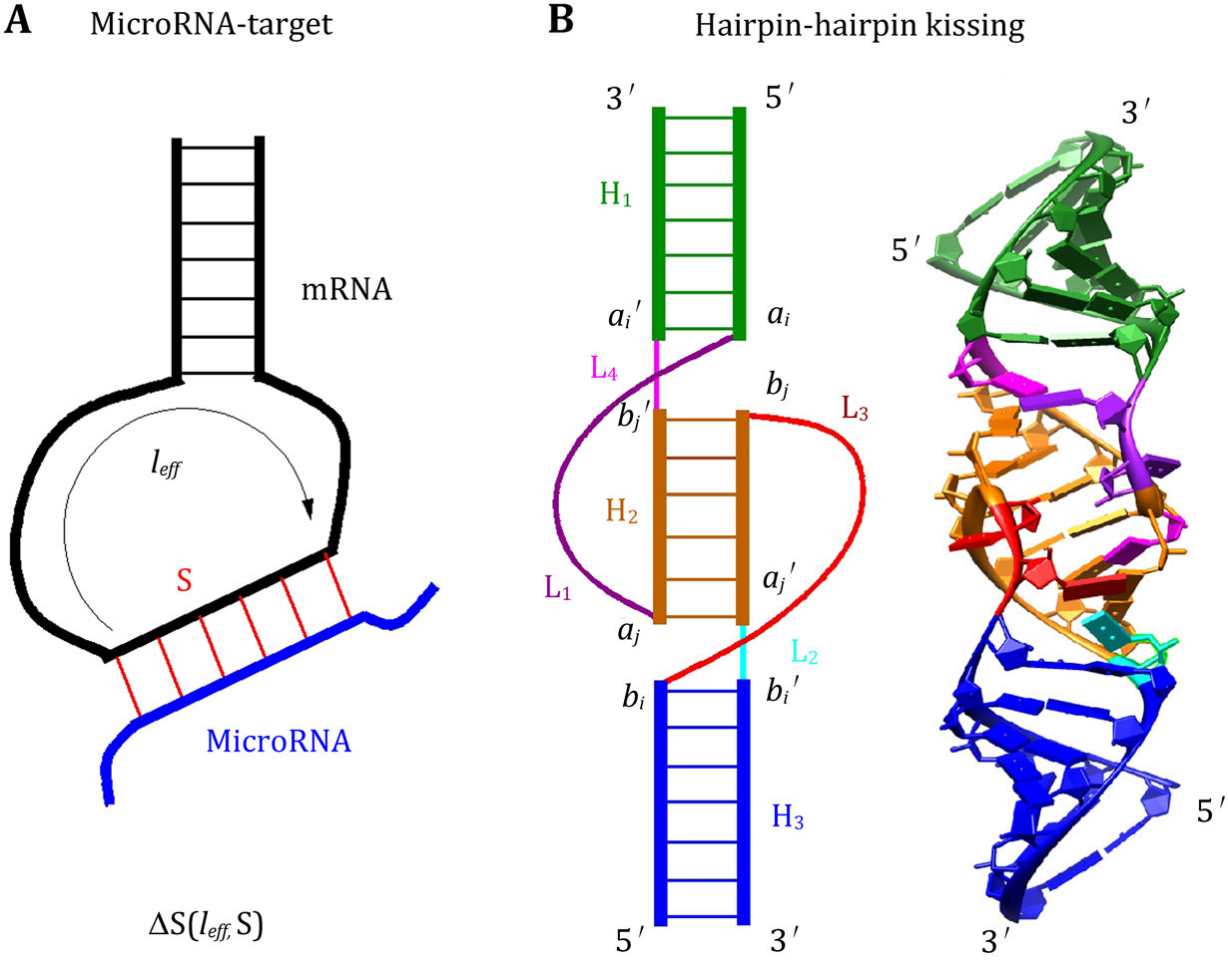
**A** A schematic figure for the microRNA–target-binding complex. The entropic change upon the binding between microRNA and the mRNA ΔS(*l*_eff_, *S*) depends on the length of the binding site *S* and the effect loop length *l*_eff_. **B** A schematic diagram and all-atom structure of the hairpin–hairpin kissing complex

Unlike the entropy (free-energy) parameters for simple loops (hairpin, bulge, and internal loops), which have been determined from thermodynamic experiments (Turner and Mathews 2010). Quantitative understanding of many other interactions remains very limited. Moreover, because of the possible conformational coupling between the different loops and between loops and helices, the loop entropies are not additive for tertiary motifs such as loop–loop kissing contacts (Fig. 1). Previous studies on the kissing complexes and other RNA folding systems such as pseudoknots suggested that a reliable estimation for the entropy is indispensable for folding predictions (Cao and Chen 2006, 2009; Andronescu et al. 2010a, b). Accurate treatment for the entropy and free energy for tertiary structure formation is a bottleneck.

Dirks and Pierce (2003) introduced a simplified energy model for H-type pseudoknots:

$$ G_{\text{pseudo}} = \beta_{1} + \beta_{2} B^{\text{P}} + \beta_{3} U^{\text{p}} , $$

where *β*_1_ is the penalty for introducing a pseudoknot, *B*^p^ is the number of base pairs that border the interior of the pseudoknot, and *U*^p^ is the number of unpaired bases inside the pseudoknot. Later, this energy model was extended (Sperschneider et al. 2011) to parameterize hairpin–hairpin kissing motifs, as shown in Fig. 1. In essence, by decoupling the interplay between helices and loops in the tertiary motifs, this energy model approximates the non-additive energy with an additive model.

Based on the polymer physics theory (de Cloizeaux 1974; Grosberg and Khokhlov 1994), Aalberts et al. (2013) proposed the following expressions (Meng and Aalberts 2013) for the free energy cost of stretching mRNA hairpin loops upon small RNA binding (Fig. 1):

$$ \beta G\left( {N,M,z} \right) = \frac{3}{2}\ln \left( {N^{\frac{6}{5}} a^{2} + M^{\frac{6}{5}} b^{2} } \right) + \beta C_{0} - \frac{5}{18}\ln \left( {\frac{z - 2.4}{{R_{\text{F}} - 2.4}}} \right) - \left( {N + M - 2} \right)\ln \left( {\frac{Na + Mb - z}{{Na + Mb - R_{\text{F}} }}} \right) + \left( {\frac{5}{{2R_{\text{F}} }} + \frac{5}{{18\left( {R_{\text{F}} - 2.4} \right)}} - \frac{N + M - 2}{{Na + Mb - R_{\text{F}} }}} \right)(z - R_{\text{F}} ), $$

where *N* is the number of single-stranded backbone segments (of length *a* = 6.2 Å), and *M* is the number of helix crossing segments (of length *b* = 15 Å). The Flory radius $R_{\text{F}} = \left( {N^{\frac{6}{5}} a^{2} + M^{\frac{6}{5}} b^{2} } \right)^{1/2}$ represents the characteristic end-to-end separation of a self-avoiding chain (Aalberts and Hodas 2010). *β* = *k*_B_*T* and the constant *C*_0_ can be set on the basis of experiment. The parameter *z* is the end-to-end separation of a helix, which can be calculated as

$$ z(s) = \left\{ {(hs)^{2} + r^{2} \left[ {1 - \cos \left( {\frac{2\pi s}{11}} \right)} \right]^{2} + r^{2} { \sin }^{2} \left( {\frac{2\pi s}{11}} \right)} \right\}^{1/2} = \left[ {(hs)^{2} + 4r^{2} { \sin }^{2} \left( {\frac{2\pi s}{11}} \right)} \right]^{1/2} $$

for an A-form RNA helix of (*s* + 1) base pairs, with *h* = 2.7 Å and *r* = 9.9 Å. We note that the freely jointed chain (FJC) model used to derive the above free energy upon small RNA binding to mRNA hairpin loops does not consider the excluded volume effect between the A-form helix and the single-stranded chain; moreover, the FJC model can only provide an estimation for long chains.

To compute entropy of hairpin, bulge/internal and multibranch loops of long length (up to 50 nt), with an efficient sampling method based on the sequential Monte Carlo principle, Zhang et al. (2008) developed optimized discrete k-state models based on RNA backbone conformations in known RNA structures. The method is general and can be applied to calculating entropy of loops with high complexity.

### 3D structure predictions

RNA 3D structure prediction is still at its early stage (Shapiro et al. 2007; Andersen 2010; Laing and Schlick 2011; Rother et al. 2011; Sim et al. 2012). Current RNA folding algorithms are generally limited to simple (short) structures, hampered by the challenges including adequate treatment of the conformational sampling and the evaluation of the energies for the tertiary contacts. Table 1 describes some of the most recently developed algorithms, ranging from coarse-grained modeling to various structure–assembly, and other conformational sampling approaches.

**Table 1:** A partial list for the computational models for RNA tertiary structure prediction and interactive manipulation

Model	Simulation method	References
YAMMP/YUP	Coarse-grained (1-bead/nt)	Wang et al. (1999) and Tan et al. (2006)
NAST	Coarse-grained (1-bead/nt)	Jonikas et al. (2009)
iFoldRNA	Coarse-grained (3-bead/nt)	Sharma et al. (2008)
CG	Coarse-grained (3-bead/nt)	Shi et al. (2014)
CG	Coarse-grained (5-bead/nt)	Xia et al. (2010, 2013)
HiRE-RNA	Coarse-grained (6- or 7-bead/nt)	Pasquali and Derreumaux (2010)
DMD	Discrete molecular dynamics	Ding et al. (2008)
RAG	Graph theory	Izzo et al. (2011) and Kim et al. (2014)
FARNA/FARFAR	Fragment assembly	Das and Baker (2007) and Das et al. (2010)
MC-Sym	Fragment assembly	Parisien and Major (2008)
Vfold	Coarse-grained (3-bead/nt) and motif-based template assembly	Cao and Chen (2011)
3dRNA	Secondary-elements assembly	Zhao et al. (2012)
BARNACLE	Probabilistic model for sampling	Frellsen et al. (2009)
RNA2D3D	Interactive manipulation	Martinez et al. (2008)
Assemble	Interactive manipulation	Jossinet et al. (2010)

#### Coarse-grained approaches

Coarse-grained representation can largely reduce the degrees of freedom and thus enhance the conformational sampling. YAMMP/YUP (Wang et al. 1999; Tan et al. 2006) and NAST (Jonikas et al. 2009) represent RNA with just one pseudo-atom per nucleotide residue: P and C3′, respectively. iFoldRNA (Sharma et al. 2008) and Vfold (Cao and Chen 2005; Shi et al. 2014) represent RNA by three pseudo-atoms per residue. Ren (Xia et al. 2010, 2013) uses 5-bead to represent each nucleotide, and HiRE-RNA (Pasquali and Derreumaux 2010) uses six or seven pseudo-atoms for purine and pyrimidine residues, respectively. Coarse-grained systems are usually modeled with knowledge-based potentials that are derived from known structures. Combined with discrete molecular dynamics (DMD) (Ding et al. 2008) or other similar methods, this approach has the potential to predict structures and folding mechanism for large RNAs. For example, a recently developed 3-bead model (Shi et al. 2014) can achieve 3.5 Å RMSD on average for 46 small RNAs including pseudoknots. Combined with Monte Carlo-simulated annealing algorithm and a coarse-grained force field with implicit salt, the model may provide reliable predictions for the stability and salt effect with the mean deviation ∼1.0 °C of melting temperatures, compared with the extensive experimental data for 30 RNA hairpins.

Another coarse-grained approach is the graph theory-based tool (RAG) (Izzo et al. 2011; Kim et al. 2014) for sampling RNA global helical topologies. RAG represents RNA 2D structure as planar tree or dual graphs to assist the cataloging, analyzing, and designing of RNA structures. With the knowledge-based potential for internal loop orientations, such as bending and torsion of internal loops, the combination of graph theory and Monte Carlo-simulated annealing sampling shows great promise for assembling global features of RNA architecture: graph RMSDs range from 2.52 to 28.24 Å for RNAs of the size 25–158 nucleotides.

#### Structure–assembly approaches

Based on the assumption that the 3D fold is more conserved and can be recognized by the alignment of sequences and secondary structure patterns, the template-based modeling (Das and Baker 2007; Parisien and Major 2008; Das et al. 2010; Cao and Chen 2011; Zhao et al. 2012) has shown promising achievements in RNA 3D structure predictions. In structure-assembly approaches, RNA 3D structures are built based on the known structures modules ranging from fragments of 1–3 nucleotides to 2D structural motifs.

FARNA/FARFAR (Das and Baker 2007; Das et al. 2010) models RNA 3D structures by assembling of short fragments (1–3 nucleotides) from a single crystal structure via a Monte Carlo procedure guided by a knowledge-based energy function that encodes base-stacking and base-pairing potentials. It can reach atomic resolution (<3.0 Å) for most short RNAs (<30 nt). MC-Sym (Parisien and Major 2008) builds all-atom structures using the 3D version of the nucleotide cyclic motif (NCM) fragments. The 3D NCM library was built from a list of 531 known RNA 3D structures. Due to the limited NCM fragments for large, complex NCM motifs, such as 6-way junctions and kissing loops, current MC-Sym is limited to short RNAs requiring 2D structures as input. 3dRNA (Zhao et al. 2012) builds the whole RNA structure from the smallest secondary elements (SSEs) by a two-step assembling procedure. The SSEs are defined as base-pair hairpin, internal/bulge loop, pseudoknot loop and junction, which are extracted from known structures.

One of the common limitations to the structure–assembly approaches is the degree of divergence of the fragment library. Given the limited number of known RNA structures, structural motif templates with the required high sequence identity are difficult to attain. The lack of reliable structural motifs for loops and junctions greatly hampers accurate 3D RNA structure prediction. Moreover, the template-based structure prediction models cannot predict structures with “new” motifs.

As for the coarse-grained models, incorporating experimental data can dramatically improve the accuracy for the structure–assembly approaches. For instance, constraints using structural inference of native RNAs by high throughput contact mapping, such as the multiplexed hydroxyl radical (–OH) cleavage analysis (MOHCA), improve the FARNA’s prediction (Das et al. 2008). For the 158-nt P4–P6 domain of the group I intron, MOHCA leads to an improvement of RMSD from 35 Å with FARNA to 13 Å.

#### Sampling algorithms

One of the challenges for current RNA structure prediction is the problem of conformational sampling. Even for the DMD with knowledge-based energy functions at different coarse-grained levels, a major issue is that sampled conformations often remain close to the initial starting model (Sim et al. 2012). The molecular system is trapped in its local energy minima for the most part of the computational time, and the barriers between local minima on the energy landscape hinder transitions between different low-energy states. To overcome this difficulty requires the use of special simulation techniques (Li and Scheraga 1987; Rahman and Tully 2002; Minary et al. 2004; Curuksu and Zacharias 2009) to achieve effective sampling of conformational space.

A probabilistic model, called BARNACLE (Frellsen et al. 2009), allows for efficient sampling of RNA conformations in continuous space and with related probabilities. Using coarse-grained base-pairing information, BARNACLE generates reasonable RNA-like structures for small RNAs (<50 nt). However, the method is mostly limited to short RNAs because of the rapid increase in complexity of the probabilistic model.

#### Interactive manipulation

Many RNA structure design algorithms, such as RNA2D3D (Martinez et al. 2008) and Assemble (Jossinet et al. 2010), are quite efficient. This interactive graphical tools are useful to analyze and build RNA architectures, but have less ability for RNA structure predictions, since they rely on manual application of expert knowledge.

## Vfold: from sequence to 3D all-atom structures

Vfold (web server (Xu et al. 2014): http://rna.physics.missouri.edu) is a model used to predict RNA 2D and 3D structures and the folding stability from the sequence. The model distinguishes itself from other models by two unique features: physics-based modeling of conformational entropy for 2D structure prediction, and template-based multiscale modeling for 3D structure prediction.

### Entropy parameters for tertiary motifs

Using the P-C4′ and C4′-P virtual bonds to represent the backbone conformations, the Vfold model (Cao and Chen 2005) samples loops/junction conformations in the 3D space through conformational enumeration (Xu et al. 2014). By calculating the probability of loop formation, the model estimates the conformational entropy parameters for the formation of the different types of loops such as pseudoknot loops and hairpin–hairpin kissing motifs. The model has the advantage of accounting for chain connectivity, excluded volume, and the completeness of conformational ensemble. Studies by us and other groups show that an accurate entropy parameter improves the prediction of RNA secondary structures and thermodynamic stabilities (Andronescu et al. 2010). Here, we use the hairpin–hairpin kissing motif to illustrate the Vfold calculation for the entropy of an RNA/RNA kissing complex.

The hairpin–hairpin kissing complex, shown in Fig. 1B, consists of three stems and four loops. We assume loop *l*_2_ and *l*_4_ are short, with ≤1 nucleotide, which favors the formation of coaxial stacking interaction between stem *H*_1_ and *H*_2_ and between *H*_2_ and *H*_3_. Therefore, the entropic cost upon the formation of loop–loop kissing *S*(*H*_2_, *l*_1_, *l*_3_) depends on the length of the stem *H*_2_ and the single-stranded loops of *l*_1_ and *l*_3_. The computation involves three steps:

1. Due to the nature of the coaxial stacking between stems of *H*_1_, *H*_2_, and *H*_3_, the relative orientation between stems of *H*_1_ and *H*_3_ is determined by the length of stem *H*_2_. The coordinates of the 8 nt ($a_{\text{i}} ,a_{i}^{{\prime }} ,a_{j} ,a_{j}^{{\prime }} ,b_{i} ,b_{i}^{{\prime }} ,b_{j} ,b_{j}^{{\prime }} ,$ shown in Fig. 1B) are adopted from the known NMR structure as the template. The final coordinates of the 8 nt for different length of *H*_2_ are generated according to the A-formed *H*_2_ and the template.
2. For each helix orientation, with well-defined (*a*_*i*_, *a*_*j*_) of the starting and ending nucleotides for the loop *l*_1_ and (*b*_*i*_, *b*_*j*_) of the starting and ending nucleotides for the loop *l*_3_, we model loop conformations as self-avoiding walks of the virtual bonds on diamond lattice (Cao and Chen 2005) to sample loops/junctions 3D conformations. The connection between the A-form helix and the discrete loop conformations is realized through an iterative optimized algorithm (Ferro and Hermans 1971).
3. A key issue in the conformational count is the excluded volume interaction between loop and helix and between the different loops. In the Vfold model, this can be explicitly taken into account by disallowing overlapping virtual bonds when the loop conformations are generated in the virtual bond diamond lattice. Assuming the interactions in the loops are weaker than the base stacking interactions that stabilize a 2D structure, we can estimate the loop entropy parameter as the logarithm of the conformational count.

**Fig. 2:**
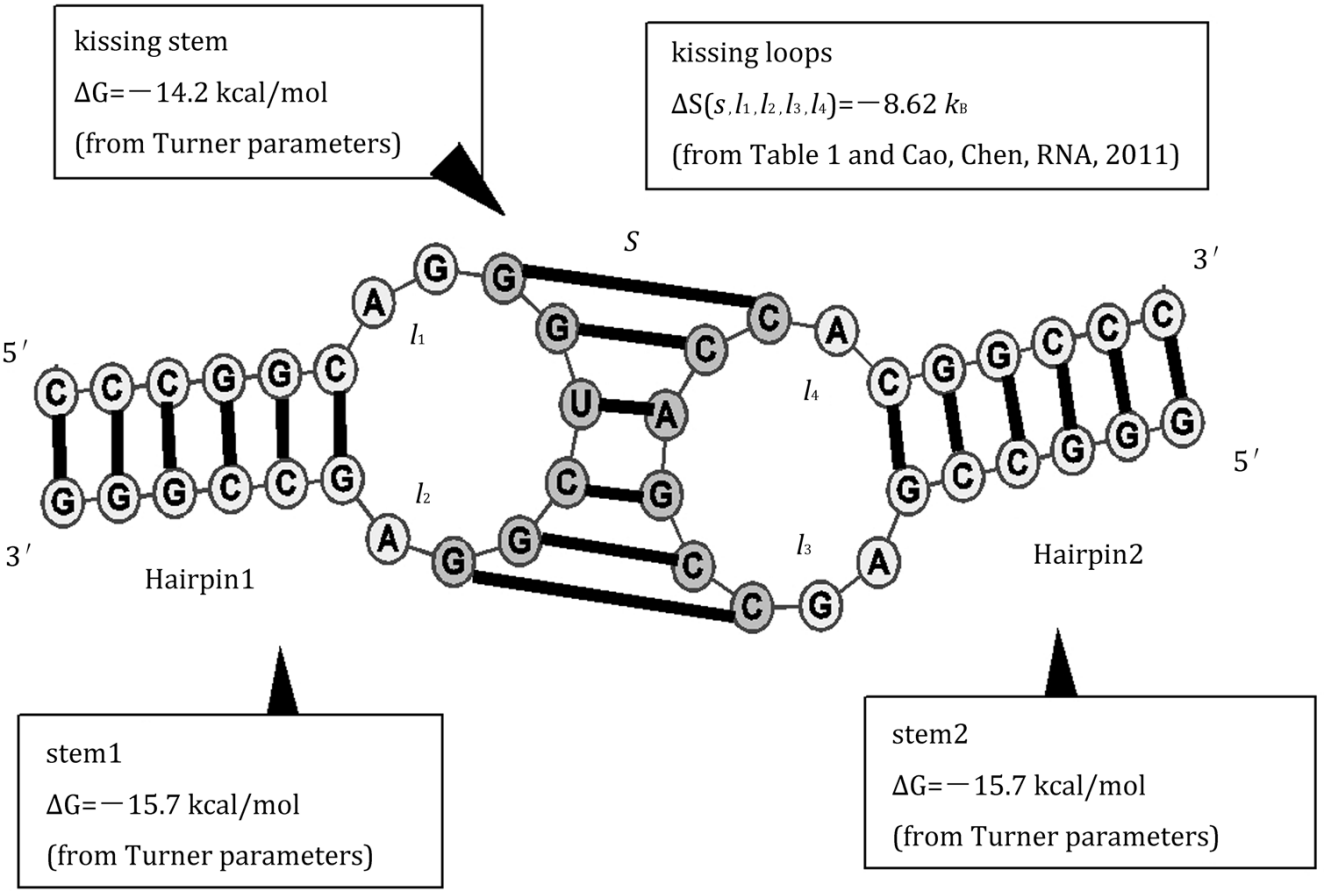
The evaluation of the free energy for a hairpin–hairpin kissing complex using the loop entropy parameters in Table 1 in Cao and Chen (2011) and the Turner parameters (Turner and Mathews 2010)

The Vfold-predicted loop entropies (Table 2) enable folding free energy calculations for RNA/RNA complex such as for microRNA–target-binding (Cao and Chen 2012) and hairpin–hairpin kissing complex systems (Cao and Chen 2011; Cao et al. 2014). For example, for the kissing complex shown in Fig. 2, the free energy is computed as

$$ \Updelta G = \Updelta G_{\text{stem1}} + \Updelta G_{{{\text{stem}}2}} + \Updelta G_{\text{kissing - stem}} - T\Updelta S\left( {S,l_{1} ,l_{2} ,l_{3} ,l_{4} } \right) = - 15.7 - 15.7 - 14.2 + 8.62(k_{\text{B}} T) = - 40.2\;{\text{kcal/mol}}, $$

where *S* is the number of base pairs in the kissing stem, and *l*_1_, *l*_2_, *l*_3_, *l*_4_ are the length of loops 1, 2, 3, and 4, respectively. The entropic energy of kissing loop is estimated by $k_{\text{B}} \ln \frac{{\omega_{6,2,2} }}{{\omega_{\text{coil}} }},$ with ${ \ln }\omega_{6,2,2}$ is read from Table 1, and ${ \ln }\omega_{\text{coil}}$ = 2.05 *l* + 0.1 (*l* is the chain length of loop *l*_1_ or *l*_3_) from the polymer physics theory.

**Table 2:** The Vfold-derived conformational entropies ${ \ln }\omega_{{H_{2} , l_{1} , l_{3} }}$ for the kissing complex for the different stem lengths and different loop lengths

*l*_3_	*H*_2_ = 3							*H*_2_ = 4						
	1	2	3	4	5	6	7	1	2	3	4	5	6	7
*l*1 = 2	−	0	0	1.8	2.6	4.2	5.8	−	1.1	0.7	1.4	3.4	5.0	6.7
*l*_1_ = 3	−	0	−	1.6	1.1	1.4	2.5	−	0.7	1.4	0.7	3.4	4.9	6.6
*l*_1_ = 4	−	1.8	1.6	3.8	4.2	5.8	7.4	−	1.4	0.7	−	2.7	4.1	5.7
*l*_1_ = 5	−	2.6	1.1	4.2	4.1	5.4	7.0	−	3.4	3.4	2.7	5.3	6.7	8.4
*l*_1_ = 6	−	4.2	1.4	5.8	5.4	6.3	7.8	−	5.0	4.9	4.1	6.7	7.9	9.5
*l*_1_ = 7	−	5.8	2.5	7.4	7.0	7.8	9.3	−	6.7	6.6	5.7	8.4	9.5	11.2

*l*_3_	*H*_2_ = 5							*H*_2_ = 6						
	1	2	3	4	5	6	7	1	2	3	4	5	6	7
*l*_1_ = 1	0	1.4	1.4	2.8	3.7	5.2	6.7	−	−	−	−	−	−	−
*l*_1_ = 2	1.4	2.8	2.4	4.1	4.8	6.3	7.8	−	0	0.7	1.1	2.2	3.3	4.7
*l*_1_ = 3	1.4	2.4	2.1	3.7	4.4	5.8	7.3	−	0.7	1.8	2.3	3.7	5.1	6.7
*l*_1_ = 4	2.8	4.1	3.7	5.4	6.1	7.6	9.0	−	1.1	2.3	2.7	4.0	5.2	6.8
*l*_1_ = 5	3.7	4.8	4.4	6.1	6.8	8.3	9.7	−	2.2	3.7	4.0	5.5	6.6	8.2
*l*_1_ = 6	5.2	6.3	5.8	7.6	8.3	9.7	11.2	−	3.3	5.1	5.2	6.6	7.6	9.2
*l*_1_ = 7	6.7	7.8	7.3	9.0	9.7	11.2	12.6	−	4.7	6.7	6.8	8.2	9.2	10.9

*l*_3_	*H*_2_ = 7							*H*_2_ = 8						
	1	2	3	4	5	6	7	1	2	3	4	5	6	7
*l*1 = 2	−	−	−	−	−	−	−	−	−	−	−	−	−	−
*l*_1_ = 3	−	−	−	−	−	−	−	−	−	−	0	2.2	4.1	6.1
*l*_1_ = 4	−	−	−	2.2	3.3	5.0	6.7	−	−	0	0.7	2.4	4.2	6.1
*l*_1_ = 5	−	−	−	3.3	4.2	6.0	7.6	−	−	2.2	2.4	3.9	5.5	7.3
*l*_1_ = 6	−	−	−	5.0	6.0	7.8	9.5	−	−	4.1	4.2	5.5	7.0	8.7
*l*_1_ = 7	−	−	−	6.7	7.6	9.5	11.2	−	−	6.1	6.1	7.3	8.7	10.4

The unit of the entropies is *k*_B_

To treat more complicated (general) loop–loop kissing complexes, as shown in the Fig. 2 of Cao et al. (2014), the stem-loop substructure’s impact on loop entropy is approximated by replacing the terminal base pairs (of the stems) by single nucleotides. Then, the effective loop lengths of *l*_1_, *l*_2_, *l*_3_, and *l*_4_ are equal to the sum of the number of unpaired nucleotides and the number of stem-loop substructures. This approximation ignores the (weak) excluded volume interference between the stem-loop substructure and the loop, thus enabling us to treat general kissing motifs.

### Template-based RNA 3D structure prediction

**Fig. 3:**
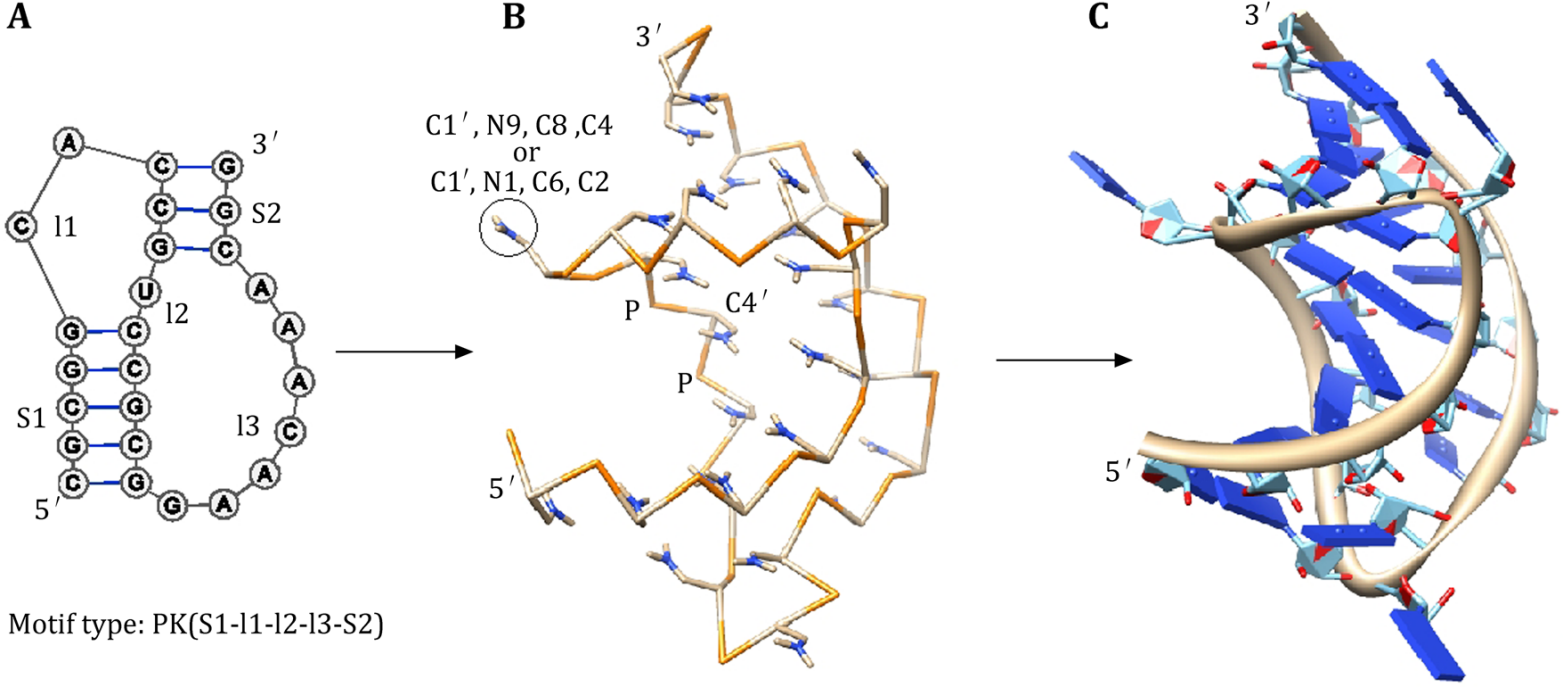
**A** The 2D structure of the BWYV pseudoknot. Vfold identifies it as a motif of “PK(5-2-1-7-3)”. **B** The virtual-bond (low-resolution) structure built from the motif-based template library. **C** The all-atom 3D structure refined by Amber energy minimization

Predicting RNA 3D structure is not a solved problem (Shapiro et al. 2007; Andersen 2010; Laing and Schlick 2011; Rother et al. 2011; Sim et al. 2012). Extensive efforts have been made to enhance the conformational sampling (Das and Baker 2007; Frellsen et al. 2009) and to establish accurate scoring functions for the ranking of the different structures (Ding et al. 2008; Parisien and Major 2008). The Vfold model can predict the 2D structures of RNA/RNA kissing complex including inter- and intramolecular base pairings. In general, a 2D structure can correspond to a large number of 3D structures due to the multiplicity of flexible loop conformations. The Vfold model-predicted virtual bond structure provides a scaffold for the construction of all-atom models of the 3D structure. The prediction of the all-atom 3D structures from a given sequence and 2D structure (base pairs) involves the following three steps (see Fig. 3):

1. To build the 3D virtual bond structure. Helices are modeled as A-form virtual-bonded helix structures. The loop/junction structures are built from the virtual bond fragments of the template structures. To identify the optimal template structure for the loops/junctions, the model screens the pretabulated template library according to the loop size (first) and the sequence (second) matches. If necessary, this step may involve sequence replacement in order to match the (same size) sequences in the template library. The model assembles the helix and loop 3D virtual-bonded structures to construct the 3D scaffold of the whole RNA.
2. To add all atoms to the virtual-bonded structure. For nucleotides in each helix, atoms are added according to the A-form helix atomic structure. The 3D conformation of the nucleotides in loops are generated by adding atoms according to the templates for base configurations, by aligning the C1′, N9, C4, and C8 for purine (A or G) or C1′, N1, C2, and C6 for pyrimidine (C or U) with those of a nucleotide in a helix. This step results in an “atomistic version” of the Vfold structure. Using three atoms instead of one atom per base, the current Vfold can better capture the base orientation from templates and also can easily replace base type accordingly.
3. Energy minimization of the whole atomistic structure using AMBER molecular dynamics simulations. The above pre-refinement structure may contain atoms/groups that clash sterically with each other. Such steric clashes can be readily resolved by the all-atom molecular dynamics simulations. With the above pre-refinement structure as the initial state, the AMBER energy minimization (Case et al. 2005) yields reliable predictions for all-atom 3D structures. In the energy minimization, the negative charges on phosphates are neutralized by Na+ cations added to the solution. The nonbonded interactions are truncated at 12 Å. Water molecules are treated by the standard TIP3P model included in AMBER software. For the most predicted structures, we found that the minimization causes only small change in the RMSD of the structure. The main advantage of the multiscale approach in the Vfold model is that the virtual-bonded tertiary structures as the initial state may already lie in the free energy basin, so the structure refinement can avoid large structural rearrangements and can thus lead to the native structure effectively.

The Vfold model predicts the 3D structure for a 2D structure based on the structural templates. To construct the template library, the model classifies the structure into different motifs, such as helices, hairpin loops, internal/bulge loops, pseudoknots, and N-way junctions (*N* ≥ 3). The motif-based template library was built from 2621 PDB structures. With the increasing number of known RNA structures, the larger and more divergent pools of the known loop/junction structures with the different types and different lengths would lead to better predictions of the 3D structure.

As shown in Fig. 3, the above strategy gives reliable predictions for the all-atom 3D structures for simple tertiary folds, such as pseudoknots and hairpin–hairpin kissing complexes. The predicted structures as a 3D scaffold will provide highly needed guidance for experiments. For example, the sequential resonance assignments from the Nuclear Overhauser Effect (NOE) data may become efficient and more accurate if the information on the nucleotide spatial proximity from the predicted (low-resolution) structure is combined with for the NMR structural determination of RNA.

## Quantitative prediction for the folding of HIV-1 DIS complex

Intermolecular loop–loop base pairing is a widespread and functionally important tertiary structure motif in RNA. Loop–loop interactions often facilitate dimerization reactions between RNA molecules. For example, in HIV-1 virus, the loop–loop kissing interaction is critical for one form of HIV-1 dimerization (Laughrea and Jette 1994; Muriaux et al. 1996; Paillart et al. 2004). In bacteria, loop–loop interaction can regulate gene expression and affect replication and translation of the bacteria (Schmidt et al. 1995; Argaman and Altuvia 2000; Repoila et al. 2003; Bossi and Figueroa-Bossi 2007; Vogel and Wagner 2007). A well-documented case is OxyS RNA repression of fhlA translation in Escherichia Coli through the formation of a stable loop-kissing interaction (Argaman and Altuvia 2000).

The dimerization process is essential for the HIV-1 replication. Muriaux et al. proposed a two-step dimerization process (Muriaux et al. 1996a, b). The kissing loop–loop complex is formed followed by a conversion to form the extended-duplex dimer due to temperature increase or protein binding. Both the kissing-loop dimer and the extended-duplex have been found in the structural measurement (Ennifar et al. 1999, 2001). Due to the lack of the thermodynamic parameters for the kissing-loop dimer, it has been difficult to determine the relative population of each dimer at the different temperature. Also, it would be biologically important to understand if the kissing-loop dimer is a kinetic intermediate or a thermodynamic stable state at room temperature. Vfold model provides a useful tool to quantitatively predict the thermodynamic stabilities for the different dimes by computing the free energy landscape of the two-stranded system (Cao and Chen 2011, 2012; Cao et al. 2014). Recently developed RNA structure prediction models are good at predicting some structures at high-accuracy resolution. For example, de novo predictive models can accurately predict the simple and short hairpin and internal loop structures (Das and Baker 2007; Ding et al. 2008; Parisien and Major 2008). However, the models cannot predict the kissing complex. The Vfold model enables the prediction of kissing complexes (Cao and Chen 2011).

Quantitative prediction of HIV-1 DIS complex requires modeling of the folding energy landscape and the structures of dimers. The partition function for the two-stranded system $Q(T) = Q_{1} \cdot Q_{2} + e^{{ - \Updelta G_{\text{associate}} /k_{\text{B}} T}} \cdot Q_{12}$ is the sum over the unbound and bound systems. Here, *Q*_1_, *Q*_2_, and *Q*_12_ are the partition functions of the (unbound) strand 1 RNA, of the (unbound) strand 2 RNA and of the kissing (bound) system, respectively. Δ*G*_associate_ is dependent on the RNA concentration *C*_T_: $\Updelta G_{\text{associate}} = \Updelta G_{\text{init}} - k_{\text{B}} T{ \ln }(C_{\text{T}} /4).$ We choose Δ*G*_init_ to be 4.1 kcal/mol according to the experimental result (Serra and Turner 1995; Zuker 2003). The calculation of *Q*_1_ and *Q*_2_ for single-stranded RNA can be achieved by many RNA secondary structure prediction models; however, the computation of *Q*_12_ requires a statistical mechanical model such as the Vfold model.

**Fig. 4:**
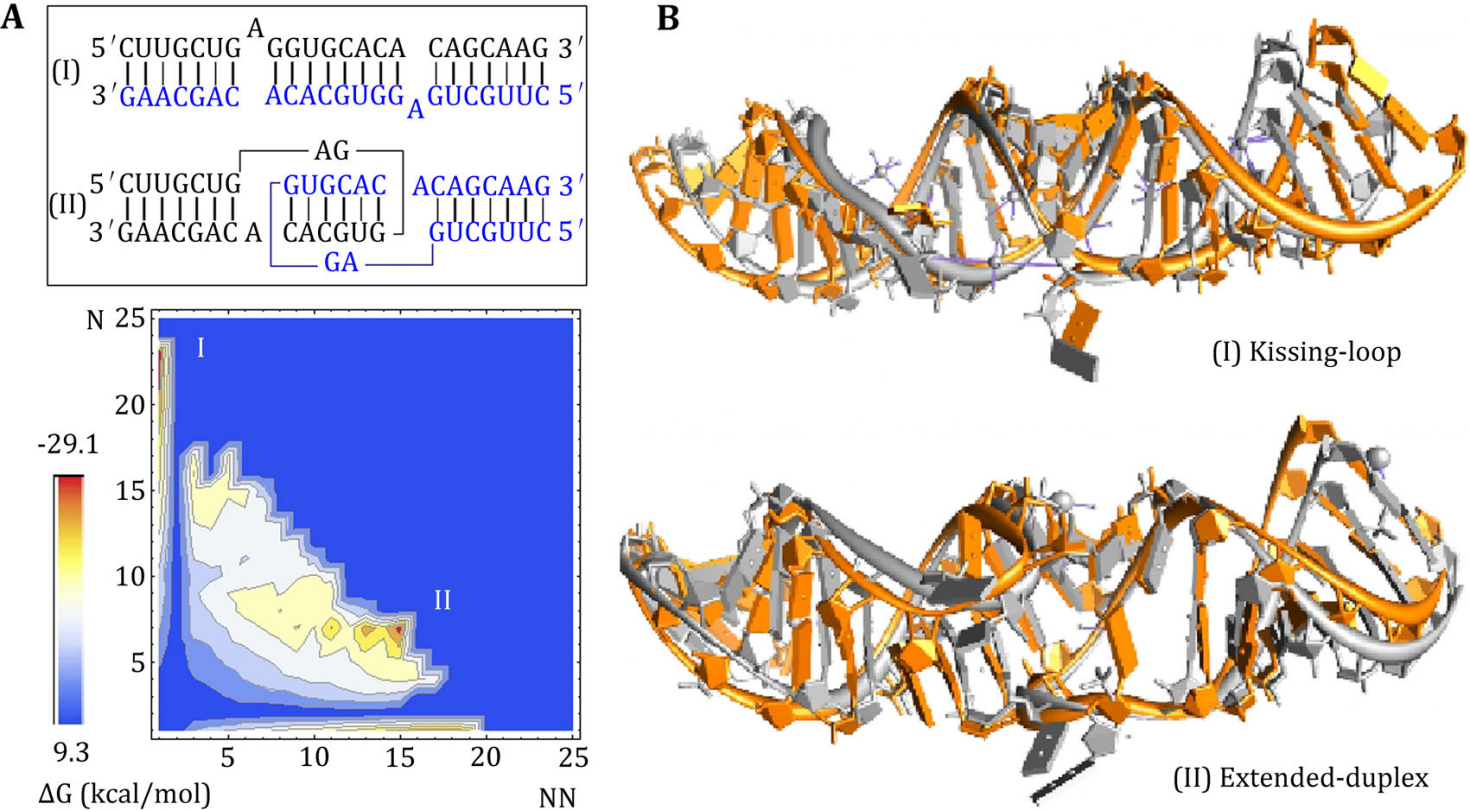
**A** The free energy landscape for the HIV-1 Mal dimer at *T* = 20 °C. The Vfold model predicts two coexisting structure (*I*, *II*), corresponding to the extended-duplex and kissing-loop dimers; respectively. In the energy landscape, *N* and *NN* are the numbers of the native and non-native base pairs, respectively. **B** The Vfold predicts 3D structures (in *orange*) for the kissing-loop and extended-duplex dimers for HIV-1 Mal dimer. The all-atom RMSDs are 3.1 and 2.9 Å with respect to the experimental structures (in *gray*) with PDB IDs 1xpe and 462d, respectively

The predicted free energy landscape for HIV-1 Mal shows two free energy minima, indicating two coexisting structures at room temperature, shown in Fig. 4A. The structural (base-pairing probability) calculations show that the free energy minima correspond to the kissing-loop dimer and the extended-duplex dimer, respectively. The extended-duplex dimer is slightly more stable than the kissing-loop dimer, with the free energy difference Δ*G* < 1.0 kcal/mol. The result suggests that the two modes of dimerization of HIV-1 Mal can coexist in thermodynamic equilibrium and can possibly interconvert with the change of the temperature and solution condition.

Moreover, based on the Vfold study, we find that the kissing-loop dimer of HIV-1 Mal is stabilized by the coaxial stacking. We built the 3D structure of the kissing-loop dimer according to the above multiscale strategy. The all-atom RMSD between predicted structure and the experiment solved NMR structure (PDB id: 1xpe) is 3.1 Å, as shown in Fig. 4B. For the extended-duplex dimer (structure I on the energy landscape), Vfold predicts the 3D structure with an RMSD of 2.9 Å (PDB id: 462d).

## Conclusions

The bottleneck for RNA tertiary structure prediction is the inability to treat the free energy, especially the entropy, for structures with long-range tertiary interactions. The virtual-bond based low-resolution conformational model (Vfold) allows us to estimate the entropy and the full free energy landscape for RNA tertiary global folds. The predicted 2D structures provide scaffolds for the construction of all-atom 3D models through molecular dynamics calculations. Validation by the experimental data for the RNA 2D and 3D structures and the folding thermodynamics as well as kinetics suggests that the statistical mechanics-based approaches can be quite reliable.

By considering the non-canonical interactions at both secondary structure and tertiary structure levels, Vfold model improves the accuracy of the secondary structure prediction and introduces more detailed constraints, besides the canonical base-pairing information, to the 3D structure modeling. However, a fast sampling algorithm balancing the completeness of the conformational space including non-canonical base-pairing information and the computational time is needed to treat large RNAs. Moreover, further development of the model should go beyond the simple hairpin–hairpin kissing complexes by estimating the entropic parameters for global folds with more complicate tertiary interactions, such as the SAM riboswitch.

With the rapidly growing size of the database of the experimentally measured RNA structures, motif template-based methods shows increasingly promising results, especially when the homologous conformations can be identified from the known structures. However, a backup plan is always needed for a good model if no known homologous conformations can be found in the PDB database. For instance, there is only a few of hairpin–hairpin kissing motifs in the current motif library. Further development of the model should address the motifs involving tertiary interactions with the ability of de novo construction.

## References

[CR1] Aalberts DP, Hodas NO (2010). A two-length-scale polymer theory for RNA loop free energies and helix stacking. RNA.

[CR2] Andersen ES (2010). Prediction and design of DNA and RNA structures. New Biotechnol.

[CR3] Andronescu MS, Pop C, Condon A (2010). Improved free energy parameters for RNA pseudoknotted secondary structure prediction. RNA.

[CR4] Andronescu MS, Condon A, Hoos HH, Mathews DH, Murphy KP (2010). Computational approaches for RNA energy parameter estimation. RNA.

[CR5] Argaman L, Altuvia S (2000). fhlA repression by OxyS RNA: kissing complex formation at two sites results in a stable antisense-target RNA complex. J Mol Biol.

[CR6] Bachellerie JP, Cavaille J, Huttenhofer A (2002). The expanding snoRNA world. Biochimie.

[CR7] Bartel DP (2009). MicroRNAs: target recognition and regulatory functions. Cell.

[CR8] Bellaousov S, Reuter JS, Seetin MG, Methews DH (2013). RNAstructure: web servers for RNA secondary structure prediction and analysis. Nucleic Acids Res.

[CR9] Bernhart SH, Hofacker IL, Will S, Gruber AR, Stadler PF (2008). RNAalifold: improved consensus structure prediction for RNA alignments. BMC Bioinformatics.

[CR10] Bindewald E, Shapiro BA (2006). RNA secondary structure prediction from sequence alignments using a network of k-nearest neighbor classifiers. RNA.

[CR11] Bossi L, Figueroa-Bossi N (2007). A small RNA downregulates LamB maltoporin in *Salmonella*. Mol Microbiol.

[CR12] Cao S, Chen S-J (2005). Predicting RNA folding thermodynamics with a reduced chain representation model. RNA.

[CR13] Cao S, Chen S-J (2006). Predicting RNA pseudoknot folding thermodynamics. Nucleic Acids Res.

[CR14] Cao S, Chen S-J (2009). Predicting structures and stabilities for H-type pseudoknots with interhelix loops. RNA.

[CR15] Cao S, Chen S-J (2011). Structure and stability of RNA/RNA kissing complex: with application of HIV dimerization initiation signal. RNA.

[CR16] Cao S, Chen S-J (2011). Physics-based de novo prediction of RNA 3D structures. J Phys Chem B.

[CR17] Cao S, Chen S-J (2012). Predicting kissing interactions in microRNA-target complex and assessment of microRNA activity. Nucleic Acids Res.

[CR18] Cao S, Chen S-J (2012). A domain-based model for predicting large and complex pseudoknotted structures. RNA Biol.

[CR19] Cao S, Xu X, Chen S-J (2014). Predicting structure and stability for RNA complexes with intermolecular loop–loop base pairing. RNA.

[CR20] Case DA, Cheatham TE, Darden T, Gohlke H, Luo R, Merz KM, Onufriev A, Simmerling C, Wang B, Woods RJ (2005). The Amber biomolecular simulation programs. J Comput Chem.

[CR21] Chen SJ (2008). RNA folding: conformational statistics, folding kinetics, and ion electrostatics. Annu Rev Biophys.

[CR22] Curuksu J, Zacharias M (2009). Enhanced conformational sampling of nucleic acids by a new Hamiltonian replica exchange molecular dynamics approach. J Chem Phys.

[CR23] Das R, Baker D (2007). Automated de novo prediction of native-like RNA tertiary structures. Proc Natl Acad Sci USA.

[CR24] Das R, Kudaravalli M, Jonikas M, Laederach A, Fong R, Schwans JP, Baker D, Piccirilli JA, Altman RB, Herschlag D (2008). Structural inference of native and partially folded RNA by high-throughput contact mapping. Proc Natl Acad Sci USA.

[CR25] Das R, Karanicolas J, Baker D (2010). Atomic accuracy in predicting and designing noncanonical RNA structure. Nat Methods.

[CR26] Deigan KE, Li TW, Mathews DH, Weeks KM (2009). Accurate SHAPE-directed RNA structure determination. Proc Natl Acad Sci USA.

[CR27] des Cloizeaux J (1974). Lagrangian theory for a self-avoiding random chain. Phys Rev A.

[CR28] Ding Y, Lawrence CE (2003). A statistical sampling algorithm for RNA secondary structure prediction. Nucleic Acids Res.

[CR29] Ding F, Sharma S, Chalasani P, Demidov VV, Broude NE, Dokholyan NV (2008). Ab initio RNA folding by discrete molecular dynamics: from structure prediction to folding mechanisms. RNA.

[CR30] Dirks RM, Pierce NA (2003). A partition function algorithm for nucleic acid secondary structure including pseudoknots. J Comput Chem.

[CR31] Ennifar E, Yusupov M, Walter P, Marquet R, Ehresmann B, Ehresmann C, Dumas P (1999). The crystal structure of the dimerization initiation site of genomic HIV-1 RNA reveals an extended duplex with two adenine bulges. Structure.

[CR32] Ennifar E, Walter P, Ehresmann B, Ehresmann C, Dumas P (2001). Crystal structures of coaxially stacked kissing complexes of the HIV-1 RNA dimerization initiation site. Nat Struct Biol.

[CR33] Ferro DR, Hermans J (1971). A different best rigid-body molecular fit routine. Acta Crystallogr A.

[CR34] Frellsen J, Moltke I, Thiim M, Mardia KV, Ferkinghoff-Borg J, Hamelryck T (2009). A probabilistic model of RNA conformational space. PLoS Comput Biol.

[CR35] Gong C, Maquat LE (2011). lncRNAs transactivate STAU1-mediated mRNA decay by duplexing with 3 UTRs via Alu elements. Nature.

[CR36] Grosberg AY, Khokhlov A (1994). Statistical physics of macromolecules.

[CR37] Gutell RR, Lee JC, Connone JJ (2002). The accuracy of ribosomal RNA comparative structure models. Curr Opin Struct Biol.

[CR38] Hajdin CE, Bellaousov S, Huggins W, Leonard CW, Mathews DH, Weeks KM (2013). Accurate SHAPE directed RNA secondary structure modeling, including pseudoknots. Proc Natl Acad Sci USA.

[CR39] Havgaard JH, Lyngso RB, Gorodkin J (2005). The FOLDALIGN web server for pairwise structural RNA alignment and mutual motif search. Nucleic Acids Res.

[CR40] He S, Su H, Liu C, Skogerbo G, He H, He D, Zhu X, Liu T, Zhao Y, Chen R (2008). MicroRNA-encoding long non-coding RNAs. BMC Genom.

[CR41] Hofacker IL (2003). Vienna RNA secondary structure server. Nucleic Acids Res.

[CR42] Hofacker IL, Fekete M, Stadler PF (2002). Secondary structure prediction for aligned RNA sequences. J Mol Biol.

[CR43] Izzo JA, Kim N, Elmetwaly S, Schlick T (2011). RAG: an update to the RNA-As-Graphs resource. BMC Bioinformatics.

[CR44] Jonikas MA, Radmer RJ, Laederach A, Das R, Pearlman S, Herschlag D, Altman RB (2009). Coarse-grained modeling of large RNA molecules with knowledge-based potentials and structural filters. RNA.

[CR45] Jossinet F, Ludwig TE, Westhof E (2010). Assemble: an interactive graphical tool to analyze and build RNA architectures at the 2D and 3D levels. Bioinformatics.

[CR46] Kertesz M, Iovino N, Unnerstall U, Gaul U, Segal E (2007). The role of site accessibility in microRNA target recognition. Nat Genet.

[CR47] Kim N, Laing C, Elmetwaly S, Jung S, Curuksu J, Schlick T (2014). Graph-based sampling for approximating global helical topologies of RNA. Proc Natl Acad Sci USA.

[CR48] Kladwang W, VanLang CC, Cordero P, Das R (2011). Understanding the errors of SHAPE-directed RNA structure modeling. Biochemistry.

[CR49] Laing C, Schlick T (2011). Computational approaches to RNA structure prediction, analysis, and design. Curr Opin Struct Biol.

[CR50] Laughrea M, Jette L (1994). A 19-Nucleotide sequence upstream of the 5′ major splice donor is part of the dimerization domain of human immunodeficiency virus 1 genomic RNA. Biochemistry.

[CR51] Leonard CW, Hajdin CE, Karabiber F, Mathews DH, Favorov OV, Dokholyan NV, Weeks KM (2013). Principles for understanding the accuracy of SHAPE-directed RNA structure modeling. Biochemistry.

[CR52] Li Z, Scheraga HA (1987). Monte Carlo-minimization approach to the multiple-minima problem in protein folding. Proc Natl Acad Sci USA.

[CR53] Liu F, Tong H, Ou-Yang Z (2006). Force unfolding single RNAs. Biophys J.

[CR54] Low JT, Weeks KM (2010). SHAPE-directed RNA secondary structure prediction. Methods.

[CR55] Martinez HM, Maizel JV, Shapiro BA (2008). RNA2D3D: a program for generating, viewing, and comparing 3-dimensional models of RNA. J Biomol Struct Dyn.

[CR56] Mathews DH, Turner DH (2002). Dynalign: an algorithm for finding the secondary structure common to two RNA sequences. J Mol Biol.

[CR57] Mathews DH, Turner DH (2006). Prediction of RNA secondary structure by free energy minimization. Curr Opin Struct Biol.

[CR58] Mathews DH, Disney MD, Childs JL, Schroeder SJ, Zuker M, Turner DH (2004). Incorporating chemical modification constraints into a dynamic programming algorithm for prediction of RNA secondary structure. Proc Natl Acad Sci USA.

[CR59] Meng Y, Aalberts DP (2013). Free energy cost of stretching mRNA hairpin loops inhibits small RNA binding. Biophys J.

[CR60] Minary P, Tuckerman ME, Martyna GJ (2004). Long time molecular dynamics for enhanced conformational sampling in biomolecular systems. Phys Rev Lett.

[CR61] Muriaux D, De Rocquigny H, Roques BP, Paoletti J (1996). NCp7 activates HIV-1Lai RNA dimerization by converting a transient loop–loop complex into a stable dimer. J Biol Chem.

[CR62] Muriaux D, Fosse P, Paoletti J (1996). A kissing complex together with a stable dimer is involved in the HIV-1 Lai RNA dimerization process in vitro. Biochemistry.

[CR63] Paillart JC, Shehu-Xhilaga M, Marquet R, Mak J (2004). Dimerization of retroviral RNA genomes: an inseparable pair. Nat Rev Microbiol.

[CR64] Parisien M, Major F (2008). The MC-Fold and MC-Sym pipeline infers RNA structure from sequence data. Nature.

[CR65] Pasquali S, Derreumaux P (2010). HiRE-RNA: a high resolution coarse-grained energy model for RNA. J Phys Chem B.

[CR66] Rahman JA, Tully JC (2002). Puddle-skimming: an efficient sampling of multidimensional configuration space. J Chem Phys.

[CR67] Repoila F, Majdalani N, Gottesman S (2003). Small non-coding RNAs, co-ordinators of adaptation processes in *Escherichia coli*: the RpoS paradigm. Mol Microbiol.

[CR68] Rother K, Rother M, Boniecki M, Puton T, Bujnicki JM (2011). RNA and protein 3D structure modeling: similarities and differences. J Mol Model.

[CR69] Sato K, Hamada M, Asai K, Mituyama T (2009). CENTROIDFOLD: a web server for RNA secondary structure prediction. Nucleic Acids Res.

[CR70] Schmidt M, Zheng P, Delihas N (1995). Secondary structures of Escherichia coli antisense micF RNA, the 5′-end of the target ompF mRNA, and the RNA/RNA duplex. Biochemistry.

[CR71] Serra MJ, Turner DH (1995). Predicting thermodynamic properties of RNA. Methods Enzymol.

[CR72] Shapiro BA, Yingling YG, Kasprzak W, Bindewald E (2007). Bridging the gap in RNA structure prediction. Curr Opin Struct Biol.

[CR73] Sharma S, Ding F, Dokholyan NV (2008). iFoldRNA: three-dimensional RNA structure prediction and folding. Bioinformatics.

[CR74] Shi YZ, Wang FH, Wu YY, Tan ZJ (2014). A coarse-grained model with implicit salt for RNAs: predicting 3D structure, stability and salt effect. J Chem Phys.

[CR75] Sim AY, Minary P, Levitt M (2012). Modeling nucleic acids. Curr Opin Struct Biol.

[CR76] Sperschneider J, Datta A, Wise MJ (2011). Heuristic RNA pseudoknot prediction including intramolecular kissing hairpins. RNA.

[CR77] Tan RK, Petrov AS, Harvey SC (2006). YUP: a molecular simulation program for coarse-grained and multiscaled models. J Chem Theory Comput.

[CR78] Turner DH, Mathews DH (2010). NNDB: the nearest neighbor parameter database for predicting stability of nucleic acid secondary structure. Nucleic Acids Res.

[CR79] Vogel J, Wagner EG (2007). Target identification of small noncoding RNAs in bacteria. Curr Opin Microbiol.

[CR80] Wang R, Alexander RW, VanLoock M, Vladimirov S, Bukhtiyarov Y, Harvey SC, Cooperman BS (1999). Three-dimensional placement of the conserved 530 loop of 16 S rRNA and of its neighboring components in the 30 S subunit. J Mol Biol.

[CR81] Wang W, Wang L, Wu J, Gong Q, Shi Y (2013). Hfq-bridged ternary complex is important for translation activation of rpoS by DsrA. Nucleic Acids Res.

[CR82] Xayaphoummine A, Bucher T, Isambert H (2005). Kinefold web server for RNA/DNA folding path and structure prediction including pseudoknots and knots. Nucleic Acids Res.

[CR83] Xia Z, Gardner DP, Gutell RR, Ren P (2010). Coarse-grained model for simulation of RNA three-dimensional structures. J Phys Chem B.

[CR84] Xia Z, Bell DR, Shi Y, Ren P (2013). RNA 3D structure prediction by using a coarse-grained model and experimental data. J Phys Chem B.

[CR85] Xu X, Chen S-J (2012). Kinetic mechanism of conformational switch between bistable RNA hairpins. J Am Chem Soc.

[CR86] Xu X, Zhao P, Chen S-J (2014). Vfold: a web server for RNA structure and folding thermodynamics prediction. PLoS ONE.

[CR87] Zhang J, Lin M, Chen R, Wang W, Liang J (2008). Discrete state model and accurate estimation of loop entropy of RNA secondary structures. J Chem Phys.

[CR88] Zhao Y, Huang Y, Gong Z, Wang Y, Man J, Xiao Y (2012). Automated and fast building of three-dimensional RNA structures. Sci Rep.

[CR89] Zuker M (2003). Mfold web server for nucleic acid folding and hybridization prediction. Nucleic Acids Res.

